# The ventrolateral preoptic nucleus: a multisystem integrator in sleep–wake regulation

**DOI:** 10.3389/fnins.2026.1773131

**Published:** 2026-05-08

**Authors:** Cong Wang, Xian Hua, Yaoxin Chen, Wenlin Xu, Zemin Wu, Yijia Wan, Xueqian Jia, Fu Xu, Xiaomei Shao, Shuo Jiang

**Affiliations:** 1 Department of Acupuncture and Moxibustion, The First Affiliated Hospital of Zhejiang Chinese Medical University (Zhejiang Provincial Hospital of Chinese Medicine), Hangzhou, China; 2 Department of Traditional Chinese Medicine, Jinhua People’s Hospital, Jinhua, China; 3 Department of Tuina, The First Affiliated Hospital of Zhejiang Chinese Medical University (Zhejiang Provincial Hospital of Chinese Medicine), Hangzhou, China; 4 Key Laboratory of Acupuncture and Neurology of Zhejiang Province, Department of Neurobiology and Acupuncture Research, The Third Affiliated Hospital of Zhejiang Chinese Medical University, Hangzhou, China

**Keywords:** GABA, galanin, neural projection, sleep, sleep–wake cycle, VLPO

## Abstract

The ventrolateral preoptic nucleus (VLPO) of the anterior hypothalamus is a major sleep-promoting region and a key component of the networks governing sleep–wake regulation. Rather than acting as a unitary “sleep center,” the VLPO is increasingly understood as a molecularly and functionally heterogeneous ensemble embedded within distributed preoptic, hypothalamic, and brainstem circuits. In this review, we revisit classical lesion, c-Fos, and electrophysiological evidence alongside recent advances in single-cell transcriptomics, projection-specific circuit mapping, and *in vivo* functional interrogation. We summarize how the core and extended VLPO and their constituent neuronal populations—including GABA/galanin co-expressing neurons, non-galanin GABAergic neurons, and glutamatergic neurons—contribute differentially to non-rapid eye movement (NREM) sleep, rapid eye movement (REM)-associated processes, state transitions, thermoregulation, and metabolic integration. We further examine how circadian timing, homeostatic sleep pressure, metabolic signals, and thermal inputs converge on VLPO-related circuitry through interactions with neighboring preoptic populations and broader hypothalamic–brainstem arousal systems. This network perspective helps reconcile classical VLPO-centered models with emerging evidence for distributed and partially redundant control of sleep–wake states. We also highlight unresolved issues, including the molecular definition of VLPO subregions, functional overlap with adjacent preoptic nuclei, inconsistencies across experimental approaches, and the challenge of translating rodent circuit findings to humans. Taken together, current evidence supports viewing the VLPO not as an isolated sleep center, but as a multisystem integrator within distributed sleep-regulatory networks.

## 1 Introduction

**Figure 1:**
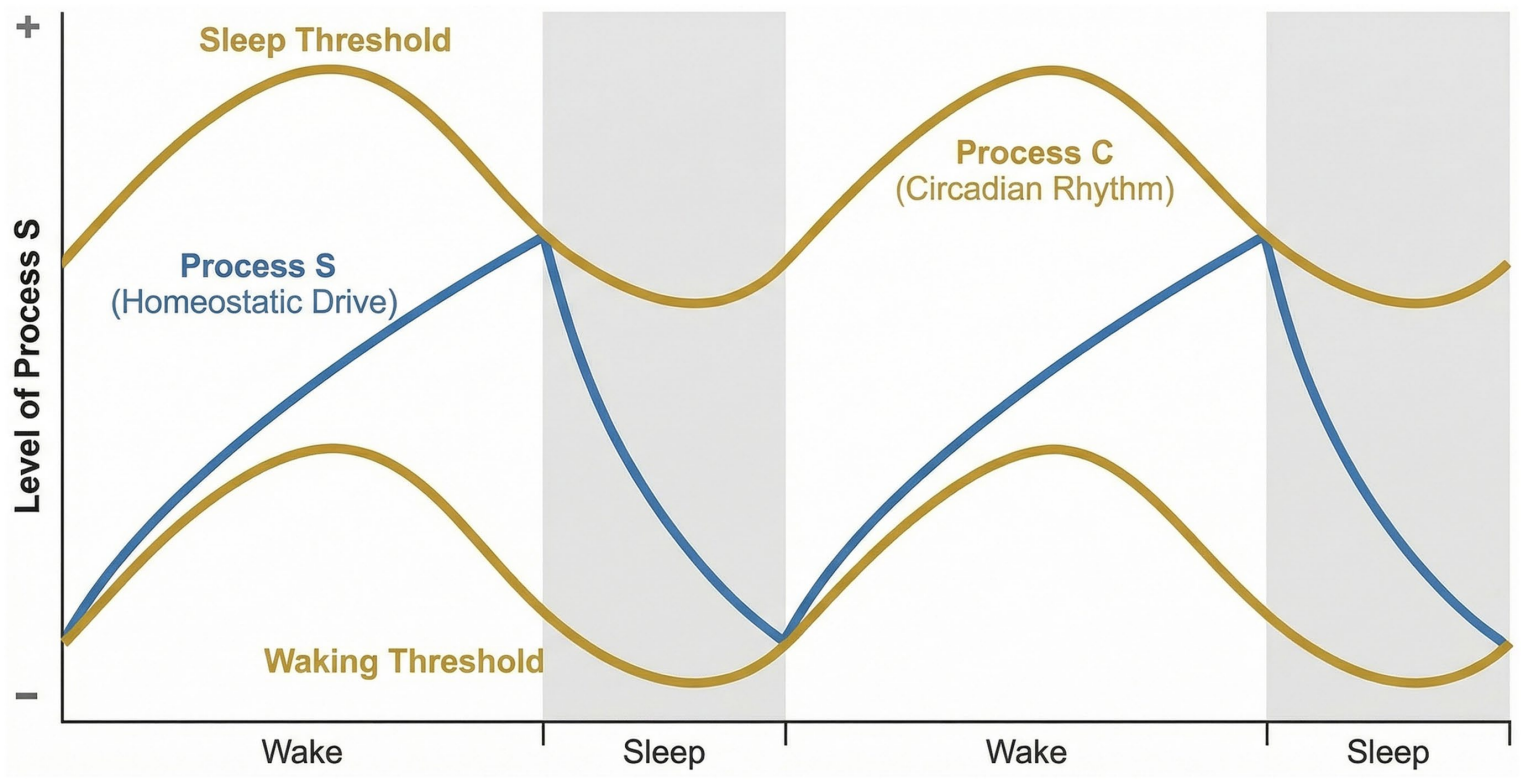
Interaction between process C and process S in sleep–wake regulation. Sleep–wake timing is governed by the interaction between the circadian process (process C) and the sleep homeostatic drive (process S). Process C, generated by the suprachiasmatic nucleus, provides rhythmic wake-promoting and sleep-facilitating signals, whereas process S reflects the accumulation and dissipation of sleep pressure across wakefulness and sleep. Sleep onset occurs when homeostatic pressure exceeds circadian arousal thresholds, and awakening occurs when circadian signals override residual sleep drive.

The sleep–wake cycle is a fundamental biological rhythm that supports cognition, brain health, immune balance, and systemic homeostasis; accordingly, its disruption is closely associated with cardiovascular disease, neurodegenerative disorders, and broader health impairment (Sarode and Nikam, 2023; Ji and Yun, 2025; Bergmann et al., 2025; Moldofsky, 1995). Mammalian sleep is organized into non-rapid eye movement (NREM) and rapid eye movement (REM) states that alternate dynamically across the day (Schwartz and Roth, 2008). These behavioral states are underpinned by interacting neural-circuit and cellular programs, including restorative and metabolic processes that become engaged during sleep (Schwartz and Roth, 2008; Mauri et al., 2022). A useful framework for understanding this organization remains the two-process model, in which Process C provides circadian timing signals that align sleep with the light–dark cycle, whereas Process S reflects the build-up and dissipation of homeostatic sleep need (Holland et al., 2018; Borbély et al., 2016; Curie et al., 2013). Importantly, Process S is best viewed as a multifactorial process rather than as the output of a single mediator: adenosine is one important component, but homeostatic sleep pressure also reflects broader activity-dependent, glial-metabolic, and sleep deprivation–related processes (Borbély et al., 2016; Curie et al., 2013; Saper et al., 2010). The interaction between Process C and Process S is illustrated in Figure 1.

At the circuit level, sleep–wake control has classically been conceptualized through the “flip-flop switch” model, in which sleep-promoting preoptic neurons and arousal-promoting systems engage in reciprocal inhibition to permit rapid and stable state transitions (Saper et al., 2010; Szymusiak and McGinty, 2008; Saper et al., 2001). This framework remains highly influential, but it is best understood as a heuristic rather than a complete description of sleep regulation. During wakefulness, monoaminergic and cholinergic arousal systems—including basal forebrain cholinergic neurons that sustain cortical activation—suppress sleep-promoting preoptic populations and stabilize the waking state (Szymusiak and McGinty, 2008; Saper et al., 2001; Zant et al., 2016). Conversely, sleep emerges when inhibitory preoptic circuitry gains functional dominance and dampens ascending arousal tone (Schwartz and Roth, 2008; Saper et al., 2010; Saper et al., 2001; Alóe et al., 2005). In this sense, physiological sleep–wake regulation is mediated by interacting hypothalamic and brainstem networks rather than by a single control center (Saper et al., 2010; Szymusiak and McGinty, 2008; Arrigoni and Fuller, 2022).

Within this broader architecture, the ventrolateral preoptic nucleus (VLPO) remains one of the best-characterized sleep-promoting regions of the anterior hypothalamus and continues to be a major target of mechanistic and pharmacological investigation (Arrigoni and Fuller, 2022; Qian et al., 2024). Over the past 25 years, convergent lesion, c-Fos, and electrophysiological studies have established that many VLPO neurons are preferentially active during sleep and contribute importantly to sleep initiation and maintenance, particularly through inhibitory GABAergic/galaninergic signaling (Arrigoni and Fuller, 2022). At the same time, growing evidence suggests that the VLPO is not functionally uniform: its neuronal populations differ in molecular identity, state dependence, and circuit engagement, indicating roles that extend beyond a simple unitary “sleep center” (Arrigoni and Fuller, 2022). This more nuanced view is especially relevant under conditions of sustained wakefulness and sleep deprivation, in which homeostatic challenge can reshape state-dependent neuronal recruitment (Arrigoni and Fuller, 2022; Yusan and Fadlilah, 2022).

Accordingly, the aim of this review is not simply to restate the classical VLPO literature, but to reposition the VLPO within a broader and more integrated framework of sleep–wake regulation (Saper et al., 2010; Saper et al., 2001; Arrigoni and Fuller, 2022). We summarize the discovery, anatomical organization, neuronal subtypes, and projection patterns of the VLPO, and then examine how VLPO-related circuits interface with circadian timing, homeostatic sleep pressure, metabolism, and thermoregulation. Particular emphasis is placed on inhibitory VLPO populations and their downstream targets, while also considering the functional relevance of non-galanin GABAergic neurons, glutamatergic neurons, and neighboring preoptic circuitry (Arrigoni and Fuller, 2022; Qian et al., 2024). By framing the VLPO as a heterogeneous integrative node embedded within distributed sleep-regulatory networks, we aim to provide a more critical and up-to-date perspective on its role in sleep–wake control and on its translational relevance to sleep-related disorders.

## 2 A brief history of the discovery of VLPO

Tracing the origins of VLPO research leads back to the 1918 influenza pandemic, a time when numerous patients developed encephalitis lethargica. Baron Constantin von Economo, an Austrian neurobiologist, carried out pathological analyses on individuals afflicted with insomnia stemming from this condition. Through postmortem examinations of these patients’ brains, he identified a strong correlation between insomnia and lesions in the anterior hypothalamus—an observation that led him to propose the presence of a “sleep control center” within this brain region (Fleming, 1930). He additionally suggested that this center induces sleep by actively suppressing the cerebral cortex and thalamus.

It was not until 1946 that Nauta undertook comprehensive, detailed investigations into the hypothalamus’s role in sleep regulation (Nauta, 1946). His experiments revealed that control lesions located dorsal to the ventral thalamus, as well as unilateral incisions in the hypothalamus, failed to induce insomnia or substantial alterations to the sleep–wake cycle. Employing a parapharyngeal surgical approach and a custom-designed hook-shaped scalpel, Nauta systematically created transverse lesions across the rat hypothalamus. His observations indicated that lesions in the caudal hypothalamus resulted in symptoms ranging from mild drowsiness to extreme sleepiness, while damage to the rostral lateral hypothalamus triggered insomnia. Based on these results, Nauta inferred that a specific hypothalamic structure—roughly overlapping with the suprachiasmatic and preoptic areas—played a critical role in mediating the ability to sleep, a structure he named the “sleep center.” He also foresightedly proposed that sleep arises from the active inhibition of the wake-promoting center by this sleep center.

A true watershed in sleep science was the discovery by Saper and colleagues that the rat VLPO is a genuine “sleep center” (Sherin et al., 1996). This previously unrecognized nucleus—defined by a clustering of “sleep-active” cells—is situated lateral to the optic chiasm, at the junction of the anterior commissure. Anatomical investigations and electrophysiological recording studies demonstrated that numerous VLPO neurons exhibit higher firing rates during sleep compared to wakefulness, with their activity escalating even further when animals recover from periods of sleep deprivation (Takahashi et al., 2009; Szymusiak and McGinty, 1989). However, accumulating evidence indicates that a subset of VLPO neurons may exhibit activity during wakefulness, reflecting functional and neurochemical heterogeneity within the nucleus, although their precise functional roles remain incompletely defined (Kostin et al., 2023; Chung et al., 2017; Lombardi et al., 2020). These electrophysiological findings are consistent with observations that VLPO neurons express the c-Fos protein during sleep but not during prolonged wakefulness (Sherin et al., 1996; Gvilia et al., 2006). Later single-cell recording experiments, however, uncovered that VLPO neurons also become active during sleep deprivation—a finding that contradicts earlier c-Fos-related research (Alam et al., 2014).

To validate the VLPO’s role in promoting sleep, Lu and his team performed targeted bilateral lesions of the VLPO region in rat models. Their results indicated an approximately 40–50% reduction in total sleep time, coupled with sleep fragmentation—effects that persisted throughout the postoperative survival period and, in some instances, for several months (Lu et al., 2000). These findings have been consistently replicated in subsequent studies, providing definitive evidence that VLPO neurons are essential for maintaining stable sleep (Arrigoni et al., 2009; Eikermann et al., 2011; Vetrivelan et al., 2012; Vetrivelan et al., 2016). Interestingly, animals with complete bilateral VLPO ablation are still capable of sleeping, suggesting that the brain harbors other sleep-promoting systems that can partially compensate for the loss of VLPO function. Additional sleep-regulating regions beyond the VLPO have also been identified. A representative example is the GABAergic parafacial zone in the rostral medulla, which has been shown to promote non-rapid eye movement (NREM) sleep through inhibitory modulation of arousal-promoting circuits (Anaclet et al., 2014; Alam et al., 2018), and the median preoptic nucleus (MnPO) (Machado et al., 2022; Anaclet and Fuller, 2017).

## 3 Location and function of the VLPO nucleus

The VLPO is located in the anterior hypothalamus, specifically within the ventral septal preoptic area adjacent to the ventrolateral part of the preoptic area (Torrealba et al., 2012; Gerstner and Yin, 2010). Anatomical studies show that VLPO projection neurons are densely distributed in the anterior hypothalamus and have direct connections with arousal nuclei such as the tuberomammillary nucleus (TMN) and locus coeruleus (LC) (Gajendra, 2023; Sherin et al., 1998). In rats, the VLPO nucleus can be specifically divided into the core area (cVLPO) and the extended area (eVLPO). Evidence suggests that the cVLPO preferentially contributes to NREM sleep regulation, whereas the eVLPO is more closely linked to REM sleep-related control (Schwartz and Roth, 2008). Recent anatomical studies have suggested that the VLPO shares overlapping cell types and functions with the medial preoptic area, linking sleep regulation with parental and sexual behaviors (Tsuneoka and Funato, 2021). The multisystem regulatory functions of the VLPO discussed in this section are summarized in Table 1.

**Table 1:** Multisystem regulatory functions of the VLPO.

Regulation system	Core function	Molecular/neural mechanism	Key pathways/targets	Data source
Sleep initiation/maintenance	Coordinate NREM/REM promotion with suppression of arousal systems	cVLPO-associated GABA/galanin neurons inhibit wake-promoting nuclei and are more closely linked to NREM promotion eVLPO-associated GABAergic pathways interact with vlPAG–LDT/PPT REM circuitry and are associated with REM-related regulation	cVLPO → TMN/LC/DR; eVLPO-associated preoptic pathways → vlPAG → LDT/PPT	Sherin et al. (1998), Kroeger et al. (2018), Lu et al. (2006), Lu et al. (2002)
Circadian rhythm	Align sleep propensity with circadian timing signals	Circadian information reaches the VLPO largely through multisynaptic hypothalamic relays, especially via SCN-connected DMH/MPOA pathways Melatonin-related signaling may further modulate VLPO-related inhibitory tone under appropriate circadian conditions	ipRGCs → SCN → DMH/MPOA → VLPO; melatonin → MT1/MT2-related preoptic modulation	Novak and Nunez (2000), Arendt (2006), Deurveilher et al. (2002), Moore (1996)
Metabolic sensing	Couple energy state to sleep propensity and arousal balance	Glucose-sensitive VLPO-related neurons increase excitability under energy-replete conditions, favoring NREM initiation Leptin- and orexin-linked hypothalamic signaling modulates VLPO-related sleep circuitry within broader metabolic-arousal networks	Glucose → KATP → VLPO-related neurons; leptin/orexin-related LHA–preoptic interactions	Scharbarg et al. (2016), Varin et al. (2015), Mavanji et al. (2015), Ramírez-Plascencia et al. (2022)
Thermoregulation	Couple sleep onset to heat loss and reduced thermogenesis	Warm-sensitive preoptic signaling biases sleep-promoting circuitry under sleep-permissive thermal conditions POA/VLPO output inhibits sympathetic premotor pathways (including the rRPa), thereby reducing brown adipose tissue thermogenesis and facilitating peripheral heat dissipation	Preoptic thermosensory pathways; POA/VLPO → rRPa → BAT	Rothhaas and Chung (2021), Conceição et al. (2019), Kroeger et al. (2018), Tan et al. (2016)

### 3.1 Functional roles of the VLPO

#### 3.1.1 Initiation and maintenance of sleep

The VLPO contributes importantly to sleep initiation and maintenance by coordinating sleep-promoting inhibition with suppression of arousal systems. Many sleep-active VLPO neurons increase firing during sleep, and VLPO-related inhibitory output targets multiple wake-promoting nuclei—including the histaminergic tuberomammillary nucleus (TMN), noradrenergic locus coeruleus (LC), serotonergic raphe nuclei, and cholinergic neurons of the basal forebrain—thereby lowering cortical arousal and stabilizing sleep states (Arrigoni and Fuller, 2022; Sherin et al., 1998; Sulaman et al., 2023). This reciprocal interaction with arousal-promoting systems forms part of the classical “flip-flop switch” framework, although current evidence suggests that such regulation is embedded within broader and partially redundant preoptic sleep networks rather than mediated by a single isolated nucleus (Lu et al., 2000; Anaclet et al., 2014; Alam et al., 2018; Machado et al., 2022; Anaclet and Fuller, 2017).

At the systems level, recruitment of VLPO-related inhibitory circuitry biases transitions from wakefulness to sleep and supports sleep continuity by reducing ascending arousal tone. Conversely, activation of wake-promoting systems suppresses VLPO-related sleep-promoting activity, reflecting a dynamic balance between competing behavioral states rather than a unidirectional switch (Moldofsky, 1995; Kostin et al., 2022; Venner et al., 2019). Detailed circuit mechanisms are discussed in Section 5.

#### 3.1.2 Regulation of circadian rhythms

The VLPO contributes to the temporal alignment of sleep with circadian timing signals, although it is not itself a primary circadian pacemaker (Riganello et al., 2019; Novak and Nunez, 2000). VLPO neuronal activity exhibits daily rhythmicity that tracks circadian organization of sleep propensity and helps link internal timing to behavioral state transitions (Riganello et al., 2019; Novak and Nunez, 2000).

Functionally, circadian influence on the VLPO is conveyed largely through indirect hypothalamic and neuroendocrine pathways rather than through a simple direct SCN-to-VLPO projection. Relay nuclei such as the dorsomedial hypothalamus (DMH) and medial preoptic area (MPOA) are thought to participate in transmitting SCN-derived timing information to sleep-promoting preoptic neurons, while melatonin-related signaling may further modulate VLPO-related inhibitory tone under appropriate circadian conditions (Novak and Nunez, 2000; Arendt, 2006; Deurveilher et al., 2002; Moore, 1996). These circadian inputs interact with homeostatic sleep pressure to shape the timing and intensity of sleep onset within broader preoptic sleep-regulatory networks. Circuit-level details are described further in Section 5.

#### 3.1.3 Metabolic integration in sleep regulation

The VLPO also participates in coupling metabolic state to sleep propensity and arousal balance. Experimental studies indicate that physiological elevations in glucose increase the excitability of subsets of VLPO sleep-promoting neurons and favor sleep, particularly NREM initiation, supporting a functional link between energy availability and sleep-promoting circuitry (Scharbarg et al., 2016; Varin et al., 2015).

In addition to glucose sensitivity, VLPO-related activity is modulated by metabolic hormones and hypothalamic arousal-metabolic signals, including leptin- and orexin-related pathways (Mavanji et al., 2015; Ramírez-Plascencia et al., 2022). These interactions suggest that metabolic regulation of sleep does not arise from a single dedicated VLPO sensor, but from broader hypothalamic–preoptic networks in which energy-replete conditions bias the system toward sleep whereas arousal-linked metabolic signals favor wakefulness. Accordingly, the VLPO is best viewed as an important integrative node linking energy homeostasis to behavioral state regulation. These metabolic influences are closely linked to thermoregulatory processes within the broader preoptic area, as discussed in Section 3.1.4.

#### 3.1.4 Thermoregulation

Thermoregulatory control is closely linked to sleep-promoting function within the broader preoptic area, including VLPO-related circuitry (Rothhaas and Chung, 2021). Physiological sleep onset is typically accompanied by a decline in core body temperature and facilitation of heat loss, indicating that thermal state is not merely a consequence of sleep but part of the permissive conditions for sleep initiation (Rothhaas and Chung, 2021). Experimental evidence further suggests that activation of preoptic/VLPO sleep-promoting neurons contributes to this transition by suppressing sympathetic premotor pathways, including those influencing the rostral raphe pallidus (rRPa), thereby reducing brown adipose tissue thermogenesis and promoting peripheral heat dissipation (Conceição et al., 2019).

Taken together, these findings support the view that thermoregulation should be considered a coupled component of VLPO-related sleep control rather than a separate auxiliary function. In this framework, sleep promotion, reduced thermogenesis, and energy conservation are coordinated across broader preoptic networks, with the VLPO acting as an important integrative node within this process (Kroeger et al., 2018). Detailed circuit mechanisms are discussed further in Section 5.

### 3.2 Cross-species comparison of the VLPO

The structure and function of the VLPO show both conservation and species-specific differences across mammals.

#### 3.2.1 Anatomical conservation and differences

##### 3.2.1.1 Rodents (rats/mice)

The VLPO is clearly divided into cVLPO and eVLPO. Dense cVLPO neurons are located lateral to the optic chiasm at the anterior commissure junction (Sherin et al., 1996; Lu et al., 2000).

##### 3.2.1.2 Humans

The homologous structure to the rodent VLPO is the intermediate hypothalamic nucleus, located in the anterior hypothalamus with similar chemoarchitectural features (e.g., GABA and galanin co-expression) (Saper, 2021). However, neuronal distribution is more dispersed and anatomical boundaries are less distinct compared to the rodent VLPO (Lim et al., 2014).

##### 3.2.1.3 Primates

Non-human primates (e.g., macaques) exhibit anatomical and neurochemical similarities to both rodents and humans, with clear cVLPO/eVLPO subdivision and GABA/galanin co-expressing neurons, but have a larger relative volume compared to total brain size (Gaus et al., 2002).

#### 3.2.2 Functional conservation

##### 3.2.2.1 Sleep-promoting role

GABA/GAL co-expressing neurons in the VLPO (or its homologous structures) promote sleep through inhibiting arousal nuclei (TMN, LC, DR) in rodents, primates, and humans (Arrigoni and Fuller, 2022; Lim et al., 2014; Gaus et al., 2002).

##### 3.2.2.2 Metabolic sensing

The VLPO’s sensitivity to glucose and leptin is conserved across species, with glucose-induced sleep promotion and leptin-mediated sleep–wake regulation observed in both rodents and non-human primates (Varin et al., 2015; Ramírez-Plascencia et al., 2022).

#### 3.2.3 Species-specific differences

##### 3.2.3.1 Neuronal subtype proportion

The proportion of glutamatergic neurons in the human intermediate hypothalamic nucleus is approximately 10–15%, higher than that in rodents (5–8%) (Saper, 2021; Vanini et al., 2020), suggesting potential species-specific differences in rapid awakening mechanisms.

##### 3.2.3.2 Circadian rhythm integration

In rodents, extensive circuit-mapping and functional studies support a prominent role of indirect SCN-to-VLPO signaling via intermediary nuclei such as the dorsomedial hypothalamus (DMH) and medial preoptic area (MPOA) in shaping circadian sleep–wake timing (Novak and Nunez, 2000; Deurveilher et al., 2002). In humans, direct evidence delineating an anatomically equivalent SCN–DMH–VLPO pathway remains limited; however, given the conserved presence of SCN outputs and hypothalamic relay nuclei, it is plausible that analogous multisynaptic routes contribute to circadian modulation of sleep-promoting regions. In parallel, evidence suggests that melatonin-mediated modulation of VLPO-homologous neurons may be relatively prominent in humans, potentially related to MT1/MT2 receptor distribution within the intermediate hypothalamic nucleus (Saper, 2021; El Dahr et al., 2022). Importantly, melatonin should be interpreted as a modulatory circadian output rather than a mutually exclusive alternative to SCN–hypothalamic relay pathways.

The human pineal gland remains functionally active and produces circadian melatonin rhythms under physiological conditions. Melatonin secretion is tightly regulated by SCN via multisynaptic sympathetic pathways (Moore, 1996; Saper, 2021), preserving its role as a principal endocrine output of the circadian system (Arendt, 2006). However, melatonin production in humans exhibits substantial interindividual variability and typically declines with aging, partly associated with pineal calcification and neuroendocrine changes. These factors may influence the relative contribution of melatonin-mediated modulation of sleep-promoting regions across individuals. Importantly, species differences in melatonin signaling between humans and rodents are more likely attributable to circadian niche (diurnal vs. nocturnal) and experimental accessibility rather than a loss of pineal functional capacity in humans. Translating rodent circuit findings to humans requires convergent validation integrating postmortem neuroanatomy, neuroimaging, and pharmacological approaches.

### 3.3 Multisystem regulatory functions of the VLPO

Table 1 summarizes four major regulatory dimensions of VLPO-related function: sleep initiation/maintenance, circadian rhythm integration, metabolic sensing, and thermoregulation. These functions should not be interpreted as independent modules. Rather, current evidence suggests that VLPO-related activity is embedded within broader preoptic networks in which circadian timing, homeostatic sleep pressure, energy status, and thermal state converge to shape sleep–wake transitions.

Accordingly, Table 1 is intended not only to condense key mechanisms and pathways, but also to highlight the multisystem coupling that underlies VLPO-related sleep regulation. Detailed mechanisms are discussed in Section 3.1, and circuit-level pathways are further elaborated in Section 5.

### 3.4 Developmental formation of VLPO circuits

The formation of ventrolateral preoptic nucleus (VLPO) circuitry arises from the broader developmental program of the anterior hypothalamus and preoptic area (Schredelseker and Driever, 2020; Fu et al., 2019). During embryogenesis, hypothalamic neurons originate from proliferative neuroepithelial zones lining the third ventricle and undergo region-specific differentiation driven by morphogen gradients and transcriptional patterning cues (Benevento et al., 2022; Corman et al., 2018). Spatial specification along the rostrocaudal and dorsoventral axes establishes functionally distinct preoptic subregions that later give rise to sleep-, thermoregulatory-, and reproductive-related nuclei, including the VLPO (Lim et al., 2014; Fu et al., 2019; Aslanpour et al., 2020).

At the molecular level, neuronal lineage specification within the preoptic and anterior hypothalamic regions is orchestrated by a coordinated network of transcription factors. Developmental regulators such as Nkx2.1, Lhx6, Dbx1, and Six3 play central roles in progenitor domain identity, neuronal migration, and neurotransmitter phenotype differentiation (Vanini et al., 2020; Munguba et al., 2021; Sandberg et al., 2018; Xu et al., 2008; Shimogori et al., 2010). Although direct lineage tracing specific to VLPO neurons remains limited, these transcriptional programs are strongly associated with the generation of inhibitory neuronal populations within the preoptic area (Kostin et al., 2022; Guo et al., 2020). Such developmental patterning is therefore thought to contribute to the emergence of GABAergic and galaninergic neuronal subtypes that later form the core sleep-promoting circuitry of the VLPO.

Postnatally, maturation of VLPO-associated circuitry parallels the progressive consolidation of sleep–wake rhythms (Gong et al., 2022). In rodent models, neonatal sleep is highly fragmented and dominated by rapid eye movement (REM)-like states, with gradual emergence of stable non-rapid eye movement (NREM) architecture across the early postnatal period (Tabuena et al., 2022; Blumberg et al., 2014). This developmental transition coincides with hypothalamic synaptic refinement and strengthening interactions between circadian and homeostatic sleep regulatory systems (Murata and Colonnese, 2019; Pandi-Perumal et al., 2025). Importantly, developmental milestones such as eye opening—marking the onset of photic entrainment via retinohypothalamic pathways—are temporally associated with the increasing circadian organization of sleep–wake behavior (Reppert and Weaver, 2002; Hoshino, 2023; Cui et al., 2025). Comparable trajectories are observed in humans, where newborn sleep is characterized by ultradian cycling and progressive circadian consolidation during infancy (Paavonen et al., 2020). Together, these observations suggest that VLPO circuit maturation is embedded within broader developmental processes linking sensory input, circadian entrainment, and inhibitory sleep network stabilization.

## 4 Neuronal types and functions in the VLPO

The main types of VLPO neurons are GABA/galanin (GAL) co-expressing neurons, non-galanin GABA neurons, and a small number of glutamatergic neurons. Glutamate decarboxylase (GAD), the synthesizing enzyme of γ-aminobutyric acid (GABA), is a core marker of GABA neurons. Studies have shown that almost all GAL immunoreactive neurons in the VLPO contain GAD immunoreactive positive neurons, and approximately 80–85% of VLPO neurons co-express GABA and GAL (Sherin et al., 1998). The dense expression of the vesicular GABA transporter (Vgat) in the VLPO further confirms the abundance of GABA-releasing neurons in this region. The major neuronal subtypes, molecular markers, projection targets, and functional characteristics are summarized in Table 2.

**Table 2:** VLPO neuronal subtypes and their characteristics.

Neuronal subtype	Molecular markers	Distribution	Key functions	Projection targets	Data source
GABA/GAL co-expressing	GAD65/67, Galanin, Vgat	cVLPO (core region), eVLPO (extended/adjacent zone)	cVLPO-associated: more closely linked to NREM promotion and inhibitory gating of arousal systems; eVLPO-associated: associated with REM-related regulation and thermoregulatory coupling	cVLPO-associated: TMN, LC, DR; eVLPO-associated: vlPAG, LDT, PPT	Sherin et al. (1998), Kroeger et al. (2018), Gaus et al. (2002), Kask et al. (1995), Pieribone et al. (1995), Xu et al. (1998), Hsieh et al. (2011), Lu et al. (2002)
Non-galanin GABA	GAD65/67, Vgat (no Gal)	Ventral/medial VLPO; VLPO-associated local GABAergic microcircuits	Wake-related local inhibition; may facilitate rapid arousal through inhibition of VLPO^Gal^ neurons and projection-specific LHA pathways	VLPO^Gal^ neurons, lateral hypothalamic area (specific downstream LHA cell types unresolved)	Arrigoni and Fuller (2022), Venner et al. (2019), Prokofeva et al. (2023)
Glutamatergic	Vglut2	Dispersed within the VLPO-associated preoptic region	Induces transient wakefulness; implicated in broader preoptic thermoregulatory and stress-related regulation	PVN, BNST, TMN(projection-specific effects remain incompletely defined)	Vanini et al. (2020), Mondino et al. (2021)

Functional assignments reflect dominant associations rather than exclusive functions, and some projection-defined effects may arise from broader preoptic networks rather than the VLPO alone.

### 4.1 GABA/GAL co-expressing neurons (VLPO^Gal^)

VLPO^Gal^ neurons are preferentially active during sleep and are inhibited by wake-associated neurotransmitters such as norepinephrine and acetylcholine through α2-adrenergic and muscarinic signaling (Gaus et al., 2002; Gong et al., 2022; Chauveau et al., 2020). Arousal signals may also indirectly suppress VLPO^Gal^ neurons by recruiting local non-galanin GABAergic feedforward inhibition. When co-released with GABA, galanin contributes to inhibitory control of wake-promoting targets including the locus coeruleus (LC), tuberomammillary nucleus (TMN), dorsal raphe nucleus (DRN), and orexin-associated lateral hypothalamic neurons (Kask et al., 1995; Pieribone et al., 1995; Xu et al., 1998; Luppi et al., 1995). Accordingly, galanin remains a widely used molecular marker of a major sleep-promoting VLPO population across rodents, primates, and humans (Gaus et al., 2002).

Notably, VLPO^Gal^ neurons are functionally heterogeneous. Core VLPO^Gal^ (cVLPO^Gal^) neurons, located within the core region of the VLPO, are more closely linked to NREM promotion, in part through inhibitory projections to histaminergic TMN neurons; lesions involving the cVLPO region can substantially reduce NREM sleep (Lu et al., 2000; Kroeger et al., 2018). Extended VLPO^Gal^ (eVLPO^Gal^) neurons, distributed dorsally and medially to the core region, show stronger associations with REM-related regulation and thermoregulatory coupling (Arrigoni and Fuller, 2022; Lu et al., 2000). REM-active neurons have been identified in the eVLPO-associated region (Szymusiak et al., 1998; Koyama and Hayaishi, 1994), and tracing studies implicate projections to pontine REM-related nuclei such as the laterodorsal tegmental nucleus (LDT) and pedunculopontine tegmental nucleus (PPT). However, because the anatomical boundaries between cVLPO and eVLPO are close and subtype-specific molecular markers remain limited, these assignments should be interpreted as dominant associations rather than fully exclusive functions.

**Figure 2:**
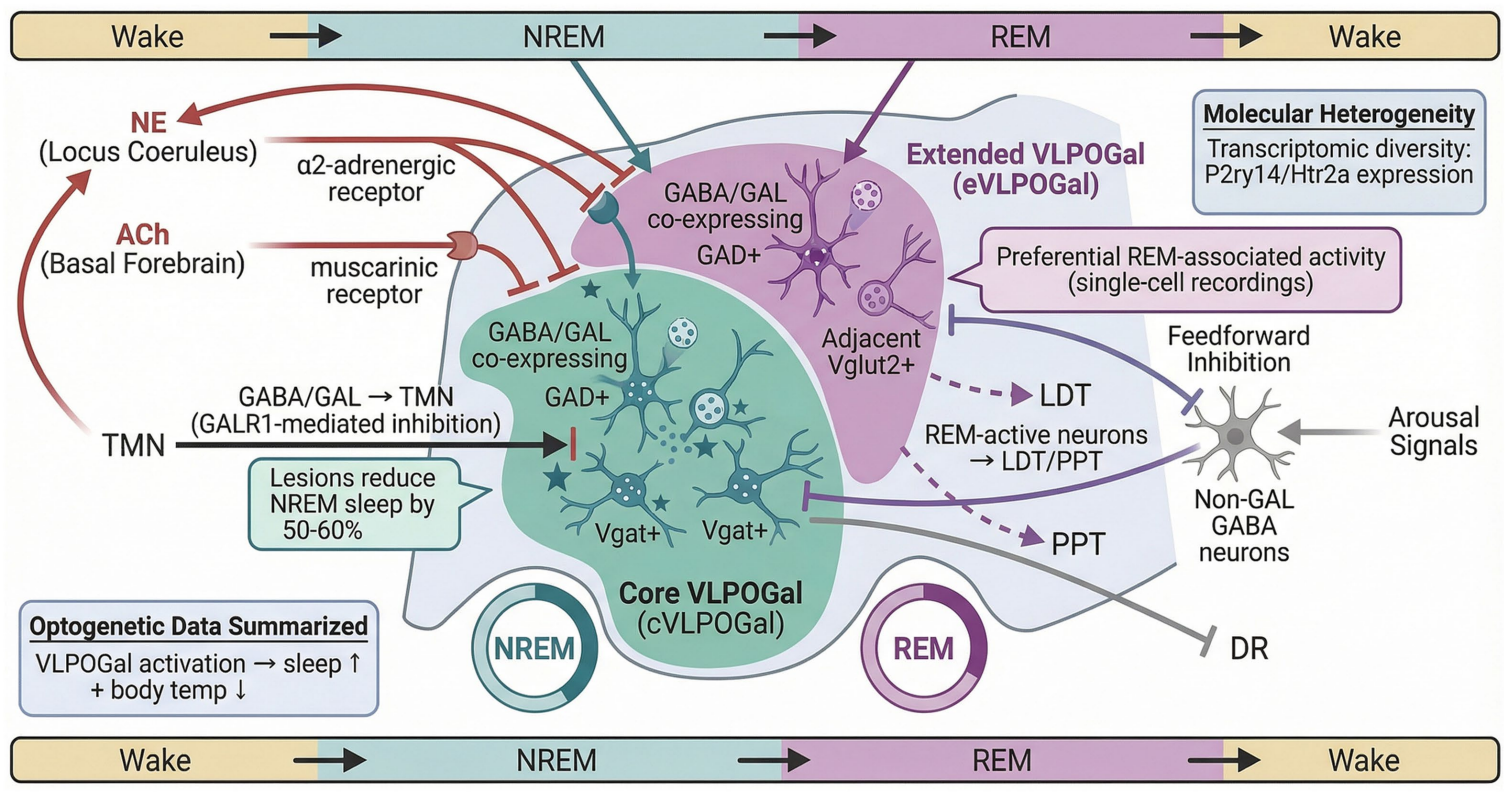
Subpopulation-specific projections and functional roles of VLPO galaninergic neurons within heterogeneous preoptic sleep-regulatory circuitry. VLPO^Gal^ neurons represent a major sleep-promoting population within the ventrolateral preoptic region, rather than the sole controller of sleep–wake transitions. Core VLPO^Gal^ neurons are more closely associated with NREM promotion through inhibitory interactions with monoaminergic arousal nuclei such as the tuberomammillary nucleus. Extended VLPOGal neurons show stronger links to REM-related circuitry and thermoregulatory coupling, including projections involving the vlPAG and LDT/PPT. Adjacent glutamatergic and non-galanin GABAergic populations, together with wake-promoting inputs from the locus coeruleus and basal forebrain, indicate that VLPOGal neurons function within a broader and heterogeneous preoptic sleep-regulatory network.

Optogenetic and chemogenetic activation of VLPO^Gal^ neurons promotes sleep and reduces body temperature in mice (Kroeger et al., 2018). Human postmortem studies further indicate that the number of GAL-positive neurons in the intermediate hypothalamic nucleus, a putative homolog of the rodent VLPO, is positively associated with sleep duration and sleep efficiency in older adults (Saper, 2021; Lim et al., 2014). Recent single-nucleus RNA sequencing studies have identified transcriptionally distinct galaninergic subpopulations, including clusters enriched for receptors such as P2ry14 and Htr2a (Guo et al., 2024). Additional transcriptomic analyses suggest state-dependent expression of endoplasmic reticulum stress–related genes and cold-inducible RNA-binding proteins in VLPO^Gal^ neurons (Guo et al., 2020). Together, these findings support the view that VLPO^Gal^ neurons represent a major, molecularly heterogeneous sleep-promoting population within broader preoptic sleep-regulatory networks (Figure 2).

### 4.2 Non-Galanin GABA neurons (VLPO^GABA^)

This subpopulation lacks galanin expression and appears to participate in wake-related microcircuits within the VLPO. Current evidence suggests that these neurons may facilitate wakefulness primarily through local feedforward inhibition of VLPO^Gal^ neurons, thereby reducing the inhibitory influence of sleep-promoting circuitry on downstream arousal systems (Arrigoni and Fuller, 2022; Venner et al., 2019). Projection-defined VLPO^GABA^ neurons targeting the lateral hypothalamic area (LHA) have also been implicated in rapid arousal responses (Venner et al., 2019; Liu et al., 2010; Prokofeva et al., 2023). However, the specific downstream LHA neuronal populations mediating this effect remain incompletely resolved. Therefore, the wake-promoting function of this subpopulation is best interpreted as arising from local inhibitory interactions and projection-specific pathways rather than from a simple, uniform circuit mechanism. These features make non-galanin GABA neurons of particular interest for understanding rapid sleep–wake transitions and VLPO microcircuit heterogeneity.

### 4.3 Glutamatergic neurons (VLPO^Vglut2^)

A smaller glutamatergic population, identified by expression of vesicular glutamate transporter 2 (Vglut2), is distributed within the VLPO-associated preoptic region (Mondino et al., 2021). Optogenetic activation of these neurons suppresses both NREM and REM sleep and induces wakefulness that typically lasts for approximately 1 hour (Chung et al., 2017; Vanini et al., 2020). Acute stimulation can rapidly convert NREM sleep to wakefulness, but the downstream circuit mechanisms underlying this effect remain incompletely defined (Chen et al., 2022; Zhang et al., 2024; Teng et al., 2022). Current evidence therefore suggests that VLPO-associated glutamatergic neurons participate in rapid arousal or state-transition control rather than in sustained wake maintenance.

Compared with canonical wake-promoting systems such as hypothalamic orexin neurons or locus coeruleus noradrenergic neurons, the wake-promoting effect of VLPO^Vglut2^ neurons appears shorter in duration (Scammell et al., 2017; Carter et al., 2010; Adamantidis et al., 2007). This temporal profile suggests that these neurons may act as transient initiators of arousal, possibly through projection-specific effects on targets such as the paraventricular hypothalamic nucleus (PVN), bed nucleus of the stria terminalis (BNST), tuberomammillary nucleus (TMN), or local preoptic microcircuits (Saper et al., 2010; Chung et al., 2017; Moffitt et al., 2018). In addition, emerging evidence links preoptic glutamatergic populations more broadly to thermoregulatory and stress-related regulation, although the extent to which these functions are specific to VLPOVglut2 neurons remains unresolved (Hrvatin et al., 2020; Tan et al., 2016).

### 4.4 GABA receptors in the VLPO

GABAergic neurons in the VLPO primarily mediate their effects through three receptor subtypes, with GABAA receptors being the dominant type. Composed mainly of α1β2γ2 and α2β3 subunits, GABAA receptors mediate fast inhibitory synaptic transmission (Wisden et al., 1992), exerting both presynaptic and postsynaptic effects: presynaptic GABAA receptors inhibit the release of neurotransmitters from wakefulness systems (e.g., TMN, LC), reducing wakefulness signal output; postsynaptic receptors mediate hyperpolarization of VLPO neurons themselves (Sallard et al., 2021; Winsky-Sommerer, 2009), forming the “flip-flop switch model” with the wakefulness system to ensure rapid transitions between sleep and wakefulness (Utami and Rahmawati, 2021).

Metabotropic GABAB receptors (GABAB1/GABAB2 heterodimers) regulate slow inhibition by inhibiting adenylate cyclase or activating G protein-coupled inwardly rectifying potassium (GIRK) channels, participating in long-term depression (LTD) in the VLPO and contributing to the long-term modulation of sleep–wake circuits (Hrvatin et al., 2020; Allada et al., 2017). Composed mainly of ρ1-ρ3 subunits, GABAC receptors are poorly expressed in the VLPO, and their specific functions remain unclear; they mediate slow inhibitory transmission by increasing Cl- influx, with potential involvement in fine-tuning sleep depth (no direct experimental evidence) (Pan et al., 2005; Gvilia, 2010). The molecular composition, signaling mechanisms, and functional roles of these receptor subtypes are summarized in Table 3.

**Table 3:** GABA receptor subtypes in VLPO and their functions.

Receptor subtype	Composition	Mechanism of action	Functional role	Data source
GABAA	α1β2γ2, α2β3	Fast inhibitory synaptic transmission; presynaptic inhibition of arousal nuclei; postsynaptic hyperpolarization	Enables rapid sleep–wake transitions; enhances VLPO inhibition of arousal-promoting system	Wisden et al. (1992), Sallard et al. (2021), Winsky-Sommerer (2009)
GABAB	GABAB1/GABAB2 heterodimers	Slow inhibition via cAMP↓ or GIRK activation	Involved in long term depression; modulates sleep homeostasis	Hrvatin et al. (2020), Allada et al. (2017)
GABAC	ρ1-ρ3 subunits	Slow Cl^−^ mediated inhibition (low expression)	Potential fine tuning of sleep depth (no direct evidence)	Pan et al. (2005)

### 4.5 Transcriptomic heterogeneity of VLPO neurons

Recent advances in single-nucleus RNA sequencing (snRNA-seq) and spatial transcriptomics have provided an unbiased molecular framework for dissecting cellular heterogeneity within the VLPO (Guo et al., 2020; Guo et al., 2024; Moffitt et al., 2018). These approaches consistently identify multiple inhibitory neuronal populations enriched for γ-aminobutyric acid (GABA)-synthetic enzymes (GAD1/2) and galanin, supporting the classical chemoarchitectural observation that sleep-promoting VLPO circuitry is dominated by inhibitory neurons. At the same time, transcriptomic data suggest that the traditional cVLPO/eVLPO and galanin/non-galanin classifications do not fully capture the molecular diversity present within VLPO-associated preoptic neurons.

Beyond confirming canonical neuronal classes, sequencing studies have revealed transcriptional subclusters that differ in receptors, ion channels, metabolic pathways, and glial-interaction signatures (Guo et al., 2020; Guo et al., 2024; Moffitt et al., 2018; Kim et al., 2025). Enrichment of purinergic signaling components, inflammatory-response genes, and astrocyte-associated markers suggests that VLPO-related sleep control is embedded in broader homeostatic, metabolic, and immune regulatory networks rather than determined by a single homogeneous sleep-promoting cell type. These molecular data therefore expand the conceptual framework of VLPO function from a regional description toward cell-type-specific and state-dependent network organization.

Importantly, transcriptomic observations currently provide stronger evidence for heterogeneity than for one-to-one mapping between molecular clusters and discrete behavioral functions. Sequencing-defined inhibitory populations are broadly consistent with sleep-active neurons identified by c-Fos mapping, optogenetic activation, and lesion studies, but direct correspondence between molecular signatures and projection-specific physiology remains incomplete (Kostin et al., 2023; Gvilia et al., 2006; Alam et al., 2014; Guo et al., 2020; Guo et al., 2024; Moffitt et al., 2018). Future integration of single-cell transcriptomics with spatial mapping, circuit tracing, and *in vivo* functional recording will be essential for linking molecular identity to physiological function within distributed preoptic sleep-regulatory networks.

## 5 Neural circuit integration of the VLPO

**Figure 3:**
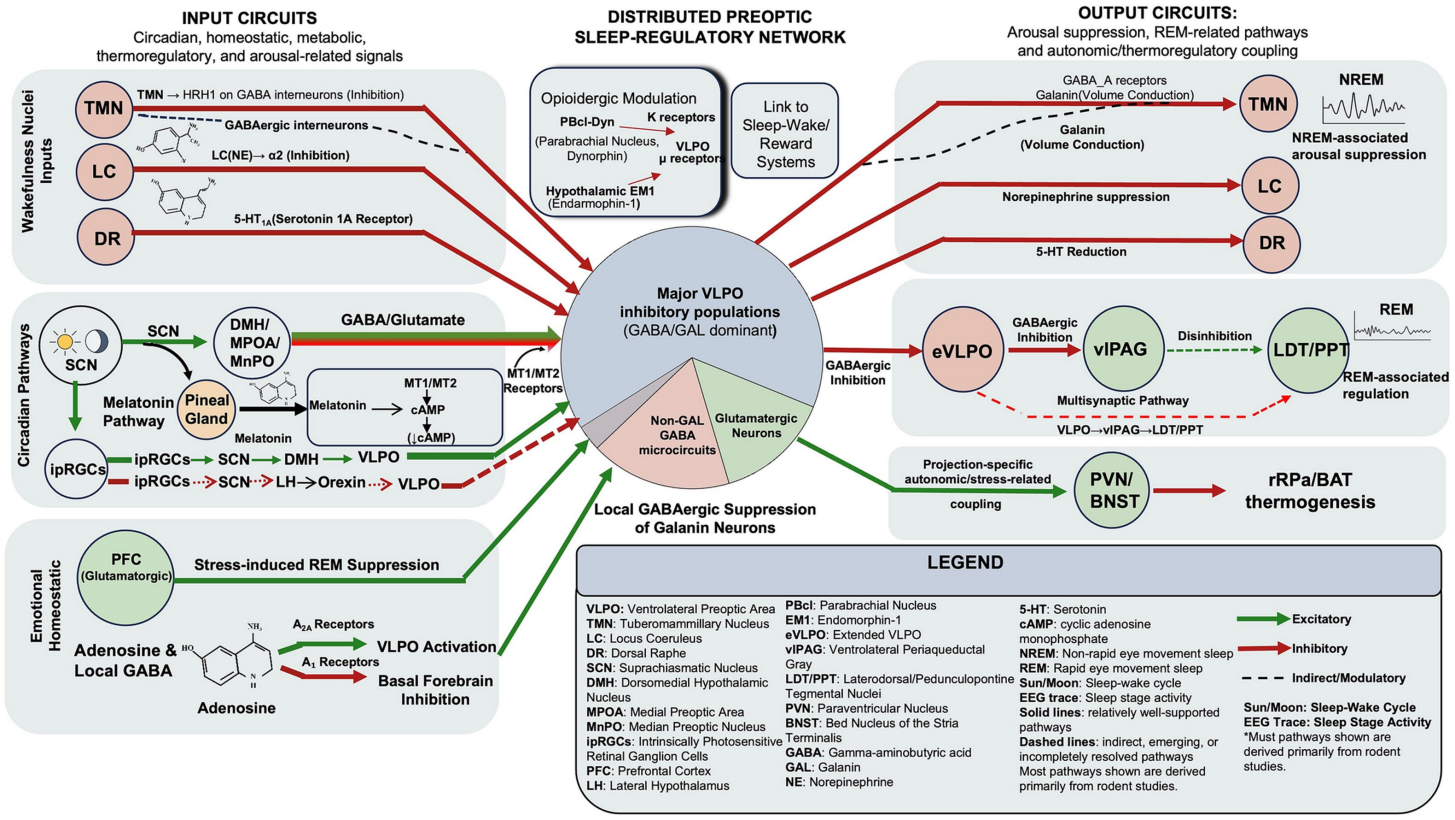
Network-level integration of VLPO circuits in sleep–wake regulation. Circadian, homeostatic, metabolic, thermoregulatory, and arousal-related signals converge on a distributed preoptic sleep-regulatory network that includes the VLPO together with DMH/MPOA/MnPO relay pathways and related hypothalamic inputs. Within this network, major VLPO inhibitory populations (predominantly GABA/galanin neurons), non-galanin GABA microcircuits, and glutamatergic neurons contribute differentially to suppression of wake-promoting nuclei (TMN, LC, and DR), REM-associated regulation through eVLPO–vlPAG–LDT/PPT circuitry, and projection-specific autonomic/thermoregulatory/stress-related outputs involving PVN/BNST and rRPa/BAT thermogenesis. Opioidergic and metabolic inputs further modulate VLPO-related activity, linking sleep–wake regulation to reward-related, homeostatic, and energy-balance processes. The schematic emphasizes that VLPO-related sleep regulation emerges from broader, partially redundant preoptic–hypothalamic–brainstem circuitry rather than from an isolated sleep center. Solid lines indicate relatively well-supported pathways, whereas dashed lines indicate indirect, emerging, or incompletely resolved connections. Most pathways shown are derived primarily from rodent studies.

Building on the functional and cellular framework outlined in Sections 3 and 4, this section examines how the VLPO participates in circuit-level control of sleep–wake states. Rather than viewing the VLPO as an isolated controller, current evidence supports a model in which molecularly and functionally distinct VLPO populations operate within distributed and partially redundant preoptic–hypothalamic–brainstem networks (Saper et al., 2010; Saper et al., 2001; Arrigoni and Fuller, 2022). Through reciprocal interactions with neighboring preoptic nuclei, hypothalamic relay regions, monoaminergic and peptidergic arousal systems, and pontine REM-related circuitry, the VLPO integrates circadian timing, homeostatic sleep pressure, metabolic state, thermoregulatory signals, and emotional or motivational influences (Machado et al., 2022; Novak and Nunez, 2000; Kroeger et al., 2018; Madrid, 2015; Lu et al., 2002; Yoshida et al., 2006). Recent transcriptomic and functional studies further indicate that these circuit functions cannot be attributed to a single homogeneous VLPO population, but instead arise from cell-type-specific and projection-related pathways (Moffitt et al., 2018; Kim et al., 2025; Mickelsen et al., 2019; Romanov et al., 2017; Kim et al., 2019). The following sections therefore summarize the principal afferent and efferent pathways of the VLPO while emphasizing their functional heterogeneity, partial redundancy, and the unresolved challenges of cross-species translation. Figure 3 schematically summarizes this updated network-based framework.

### 5.1 Defining sleep and wakefulness in preclinical animal models

Accurate interpretation of VLPO circuit function in preclinical studies requires clear operational definitions of sleep and wakefulness. In rodent models, behavioral observation alone is insufficient to distinguish vigilance states; therefore, electroencephalography (EEG) and electromyography (EMG) are routinely used for classification. NREM sleep is characterized by high-amplitude, low-frequency activity with reduced muscle tone, whereas REM sleep shows low-amplitude, theta-dominant cortical activity with near-complete muscle atonia. Wakefulness is defined by desynchronized low-amplitude EEG activity together with sustained EMG tone (Brown et al., 2012).

These distinctions are important because VLPO-related activity should be interpreted primarily in the context of physiologically regulated sleep–wake states rather than behavioral immobility alone. Many sleep-active VLPO neurons show increased firing or c-Fos expression during natural sleep (Sherin et al., 1996), whereas quiet wakefulness, general anesthesia, and pathological unconsciousness may resemble sleep behaviorally or electrophysiologically without engaging identical underlying circuitry (Joyce et al., 2025; Luo et al., 2023; Brown et al., 2010). Distinguishing these states is therefore essential for accurate interpretation of VLPO function in circuit-level studies.

### 5.2 Input circuits of the VLPO

Rather than receiving a single class of sleep-related input, the VLPO is regulated by several partially overlapping signal streams. Wake-promoting monoaminergic systems suppress sleep-active preoptic neurons and stabilize arousal; circadian relay nuclei determine temporal gating; homeostatic, metabolic, and thermoregulatory signals bias the network toward sleep when physiological conditions become permissive; and emotional or motivational influences further modulate these transitions according to behavioral context. For conceptual clarity, the principal input pathways are organized below by signal class, although many of these pathways converge on shared preoptic microcircuits rather than acting independently.

#### 5.2.1 Direct inhibitory gating by wake-promoting nuclei

Wakefulness nuclei regulate VLPO neurons primarily through monoaminergic inhibitory inputs: histaminergic neurons in the TMN indirectly inhibit VLPO activity by activating HRH1 receptors on GABAergic interneurons within the VLPO, maintaining low VLPO activity during wakefulness (Sherin et al., 1998; Liu et al., 2010; Khouma et al., 2026); noradrenergic neurons in the LC directly inhibit VLPO neurons through α2 receptors, and serotonergic neurons in the DR directly inhibit VLPO neurons through 5-HT1A receptors, thereby stabilizing wakefulness and maintaining low activity of sleep-promoting preoptic neurons (Schwartz and Roth, 2008; Wagner-Altendorf et al., 2019; Sangare et al., 2016). The monoaminergic-opioid system also projects to the VLPO, with Dynorphin (Dyn)-expressing neurons in the central lateral parabrachial nucleus (PBcl) and enkephalin (EM1)-expressing neurons in the hypothalamus directly projecting to the VLPO, regulating sleep–wake transitions through *κ* and *μ* opioid receptors, respectively (Greco et al., 2008). Specifically, κ-opioid receptor signaling modulates the activity of sleep-active VLPO neurons, which is closely related to the regulation of sleep–wake balance and reward-related behaviors (Bruijnzeel, 2009).

#### 5.2.2 Circadian relay input

The VLPO receives circadian timing information primarily through multisynaptic pathways rather than direct projections from the suprachiasmatic nucleus (SCN), the master circadian pacemaker (Novak and Nunez, 2000). As outlined functionally in Section 3, these indirect pathways provide the mechanistic substrate through which circadian signals regulate sleep-promoting VLPO activity.

Within the canonical relay circuitry, SCN output is transmitted to intermediary hypothalamic nuclei, most prominently the dorsomedial hypothalamus (DMH) and medial preoptic area (MPOA), via glutamatergic and GABAergic signaling. The DMH functions as a critical integrative hub that coordinates circadian timing signals with metabolic and autonomic inputs. From the DMH, circadian information is conveyed to the VLPO primarily through GABAergic projections, exerting phase-dependent inhibitory modulation of sleep-promoting neurons (Riganello et al., 2019; Deurveilher et al., 2002).

Through this hierarchical relay architecture, circadian signals are translated into temporally structured modulation of VLPO activity, thereby aligning sleep initiation and maintenance with the circadian cycle. Most of these relay relationships have been delineated in rodent models, and the corresponding systems-level organization in humans remains incompletely resolved.

#### 5.2.3 Convergence of homeostatic, metabolic, and thermoregulatory signals

Homeostatic sleep drive reaches the VLPO through both neuromodulatory and local circuit mechanisms. Adenosine, generated in association with prolonged wakefulness and glial-metabolic activity, promotes sleep in part by activating A2A receptor-dependent pathways that increase the excitability of sleep-promoting preoptic neurons (Choi et al., 2022; Kumar et al., 2013). Recent evidence further suggests that astrocyte-derived purines can bias VLPO microcircuits toward sleep by disinhibiting galaninergic projection neurons, providing a cellular link between wake-dependent energy consumption and sleep onset (Scharbarg et al., 2016).

Metabolic state engages the same circuitry at several levels. Physiological elevations of glucose excite subsets of VLPO sleep-promoting neurons, likely through KATP-related membrane mechanisms, thereby favoring NREM initiation when energy availability is sufficient (Scharbarg et al., 2016; Varin et al., 2015). Leptin-related signaling may further shift this balance toward sleep, at least partly through interactions with lateral hypothalamic arousal/metabolic circuits (Ramírez-Plascencia et al., 2022; Yoshida et al., 2006). Conversely, orexinergic activity opposes VLPO recruitment, coupling wakefulness to ongoing energy expenditure, motivated behavior, and arousal stability (Mavanji et al., 2015; Ramírez-Plascencia et al., 2022).

Thermoregulatory signaling converges on this homeostatic-metabolic framework rather than acting as a separate process. Warm-sensitive neurons in the MnPO and MPOA, together with peripheral thermosensory afferents relayed through spinal and brainstem pathways, provide the preoptic area with ongoing information about ambient and core temperature (Machado et al., 2022; Kroeger et al., 2018). When thermal conditions are sleep-permissive, these preoptic signals favor activation of VLPO-related sleep circuitry while simultaneously reducing the drive to cold-defense pathways. In this way, homeostatic sleep pressure, energy status, and thermal information converge on overlapping preoptic microcircuits that bias the organism toward sleep, reduced thermogenesis, and energy conservation.

Importantly, many of these signals are represented across the broader preoptic area, so their effects should be interpreted as network-level regulation with the VLPO as a major, but not solitary, integrative node.

#### 5.2.4 Emotional and motivational modulation

Emotional and motivational context can modify VLPO recruitment through forebrain and hypothalamic pathways, but these inputs are less well resolved than the canonical arousal and circadian circuits. Glutamatergic influences from prefrontal and limbic regions, together with stress-related hypothalamic inputs, may alter sleep architecture by changing the excitability threshold of preoptic sleep-promoting neurons (Chouvaeff et al., 2025; Ma et al., 2023). These influences likely interact with orexinergic and monoaminergic systems rather than constituting wholly independent VLPO pathways. Accordingly, current evidence supports a modulatory role for affective and cognitive state in VLPO function, while the precise cell-type-specific and synapse-specific routes remain incompletely defined.

### 5.3 Output circuits of the VLPO

VLPO outputs are best considered in two interacting functional layers: a classical inhibitory layer that suppresses arousal systems and stabilizes sleep, and a broader autonomic-metabolic layer that couples sleep onset to reduced thermogenesis and energy expenditure. Additional projection-specific pathways participate in REM regulation and in glutamatergic or stress-related state transitions. This organization emphasizes that VLPO function emerges through distributed circuit interactions rather than through a single unitary output channel.

#### 5.3.1 Inhibitory control of arousal systems

Sleep-promoting VLPO neurons, especially GABA/galanin populations, project to multiple wake-promoting nuclei including the tuberomammillary nucleus (TMN), locus coeruleus (LC), dorsal raphe (DR), basal forebrain, and orexin-associated lateral hypothalamic circuit (Ma et al., 2023; Cheng et al., 2020; De Luca et al., 2022). Inhibition of these targets lowers ascending arousal tone and stabilizes NREM onset and maintenance. Rather than functioning as a single on–off switch, this output pattern is better viewed as distributed inhibitory gating: different VLPO populations may engage different arousal nodes with partially overlapping functions, helping to explain why VLPO lesions produce severe but incomplete insomnia (Lu et al., 2000; Arrigoni et al., 2009; Eikermann et al., 2011; Vetrivelan et al., 2012; Vetrivelan et al., 2016). This framework also accommodates functional redundancy with other sleep-promoting preoptic populations.

#### 5.3.2 Coupling of sleep promotion to metabolic and thermoregulatory effectors

Beyond suppressing arousal, VLPO-related preoptic output couples behavioral state to autonomic physiology. One major mechanism involves inhibition of thermogenic pathways that normally support wakefulness and energy expenditure. Preoptic/VLPO activity is associated with suppression of sympathetic premotor drive to the rostral raphe pallidus (rRPa), leading to reduced brown adipose tissue thermogenesis and facilitation of heat loss (Rothhaas and Chung, 2021; Conceição et al., 2019; Kroeger et al., 2018). This physiological shift is especially relevant at sleep onset, when declining core body temperature and reduced metabolic expenditure create conditions that are permissive for NREM sleep.

Interactions with lateral hypothalamic orexin circuits add a metabolic dimension to this output architecture. By counteracting orexin-linked arousal and energy mobilization, VLPO-related inhibitory signaling helps coordinate sleep with energy conservation and reduced behavioral activation (Yoshida et al., 2006). In this framework, sleep promotion is not simply the absence of wakefulness, but an actively organized physiological state coupled to decreased sympathetic thermogenesis, lower metabolic demand, and altered neuroendocrine balance.

It should be noted, however, that these thermoregulatory and metabolic outputs are shared with adjacent MnPO/MPOA populations, and some downstream effects may arise from broader preoptic ensembles rather than the VLPO alone. Thus, the most defensible interpretation is that the VLPO participates in—rather than solely commands—a preoptic network that links sleep promotion to reduced thermogenesis and autonomic downscaling.

#### 5.3.3 Regulation of REM sleep via pontine centers

A subset of VLPO-associated neurons, particularly within the extended VLPO (eVLPO), has been implicated in REM-related control through interactions with the ventrolateral periaqueductal gray (vlPAG) and pontine cholinergic nuclei such as the laterodorsal tegmental nucleus (LDT) and pedunculopontine tegmental nucleus (PPT) (Szymusiak et al., 1998; Koyama and Hayaishi, 1994; Hsieh et al., 2011; Lu et al., 2002). In the classical formulation, inhibition of vlPAG GABAergic REM-off neurons disinhibits pontine REM-promoting circuitry, thereby facilitating REM expression (Madrid, 2015; Lu et al., 2002). Although this framework is supported by tracing and functional studies, the precise anatomical and molecular boundaries between eVLPO REM-related neurons and neighboring preoptic populations remain incompletely resolved. REM control should therefore be viewed as a projection-specific contribution of selected VLPO-associated neurons rather than as a uniform function of the entire nucleus.

#### 5.3.4 Glutamatergic and stress-related output pathways

A smaller set of VLPO neurons, including Vglut2-expressing cells and projection-defined populations targeting regions such as the paraventricular hypothalamic nucleus (PVN) and bed nucleus of the stria terminalis (BNST), indicates that VLPO output is not purely inhibitory nor uniformly sleep-promoting (Chung et al., 2017; Kostin et al., 2022; Vanini et al., 2020). Acute activation of VLPOVglut2 neurons can elicit transient wakefulness, whereas other VLPO projection pathways, such as VLPO-to-PVN circuits, have been associated with NREM-promoting or autonomic effects (Chung et al., 2017; Kostin et al., 2022; Vanini et al., 2020). These apparently divergent findings likely reflect cell-type-specific and target-specific organization rather than contradiction. Functionally, they suggest that the VLPO contains specialized subcircuits capable of coupling sleep–wake transitions to stress responsiveness, neuroendocrine regulation, and autonomic or thermoregulatory adjustments. Defining the transmitter identity and downstream targets of these subcircuits remains an important unresolved question.

## 6 Discussion and future perspectives

### 6.1 Integrative advances in VLPO sleep regulation

Over the past three decades, the ventrolateral preoptic nucleus (VLPO) has remained a major reference point for understanding sleep-promoting circuitry, because lesion, c-Fos, and electrophysiological studies consistently indicate that disruption of this region impairs sleep continuity whereas recruitment of VLPO-related neurons promotes sleep (Lu et al., 2000; Arrigoni et al., 2009; Eikermann et al., 2011; Vetrivelan et al., 2012; Vetrivelan et al., 2016). At the same time, the cumulative literature no longer supports viewing the VLPO as an isolated or unitary “sleep center.” Complete bilateral lesions produce profound but incomplete insomnia, and the existence of additional sleep-promoting populations in other preoptic and brainstem regions, including the median preoptic nucleus (MnPO) and parafacial zone, indicates that sleep–wake control is distributed and partially redundant rather than monopolized by a single nucleus (Lu et al., 2000; Arrigoni et al., 2009; Eikermann et al., 2011; Vetrivelan et al., 2012; Vetrivelan et al., 2016; Anaclet et al., 2014; Alam et al., 2018; Machado et al., 2022; Anaclet and Fuller, 2017).

Interpretation of VLPO function is further refined by cellular and circuit heterogeneity. GABA/galanin co-expressing populations remain the most extensively characterized sleep-promoting neurons, with cVLPO-associated neurons contributing prominently to NREM promotion and eVLPO-associated neurons showing stronger links to REM-related regulation and thermoregulatory coupling (Arrigoni and Fuller, 2022; Kroeger et al., 2018; Guo et al., 2024; Lu et al., 2002). At the same time, non-galanin GABAergic neurons and Vglut2-expressing neurons indicate that the VLPO contains local microcircuits and projection-defined subpopulations that participate in rapid state transitions, stress-related regulation, and autonomic coupling rather than exerting a single uniform sleep-promoting function (Chung et al., 2017; Kostin et al., 2022; Vanini et al., 2020; Liu et al., 2010). Notably, classical c-Fos mapping and later electrophysiological recordings are not fully congruent, as some VLPO neurons remain active during sleep deprivation or wake-related conditions, underscoring state-dependent and subtype-dependent diversity within the region (Sherin et al., 1996; Gvilia et al., 2006; Alam et al., 2014).

Recent transcriptomic and circuit-level studies strengthen this revised view. Single-cell and spatial approaches identify multiple inhibitory VLPO subclusters with distinct receptor, metabolic, and glial-interaction signatures, while astrocyte-derived purinergic signaling, glucose sensitivity, leptin-related modulation, and orexin-opposed arousal drive collectively demonstrate that sleep-promoting VLPO circuitry is embedded within broader homeostatic and metabolic control systems (Scharbarg et al., 2016; Varin et al., 2015; Ramírez-Plascencia et al., 2022; Guo et al., 2020; Guo et al., 2024; Moffitt et al., 2018; Kim et al., 2025). Likewise, circadian signals are conveyed largely through indirect hypothalamic relays rather than by a simple direct SCN-to-VLPO pathway, and warm-sensitive preoptic pathways couple sleep onset to heat loss and reduced thermogenesis (Novak and Nunez, 2000; Deurveilher et al., 2002; Rothhaas and Chung, 2021; Conceição et al., 2019; Kroeger et al., 2018). Together, these findings argue that the VLPO is best conceptualized as a heterogeneous preoptic node through which circadian, homeostatic, metabolic, and thermoregulatory information is integrated into sleep–wake state control.

Accordingly, the major advance in the field is not merely the expansion of VLPO-related circuitry, but a conceptual shift in how the nucleus is understood. The most defensible synthesis is that the VLPO serves as an important—but not solitary—integrative component of distributed preoptic–hypothalamic–brainstem sleep networks. Key unresolved questions include the molecular definition of cVLPO and eVLPO boundaries, the projection-specific roles of non-galanin GABAergic and glutamatergic neurons, the relative contributions of GABA and galanin to sleep promotion, and the extent to which rodent-defined microcircuits generalize to human hypothalamic sleep regulation (Saper, 2021; Moffitt et al., 2018; Kim et al., 2025; Mickelsen et al., 2019; Romanov et al., 2017; Kim et al., 2019). Addressing these issues will require coordinated use of transcriptomics, spatial mapping, projection-specific manipulations, and *in vivo* functional recording.

### 6.2 Translational and clinical implications

The translational relevance of VLPO research is supported most strongly by human neuropathological evidence. Postmortem studies show that neuron number within the ventrolateral preoptic/intermediate nucleus correlates with sleep consolidation in older adults, and similar degeneration has been associated with sleep fragmentation in Alzheimer’s disease (Saper, 2021; Lim et al., 2014). These observations suggest that disruption of VLPO-related sleep-promoting populations contributes to age- and disease-associated sleep instability, although the available human data remain largely correlative and do not yet establish cell-type-specific causality (Lim et al., 2014; Holth et al., 2017).

Preclinical experiments nevertheless provide proof of principle that VLPO-related circuits are therapeutically relevant. Activation of galaninergic sleep-promoting neurons improves sleep continuity and promotes heat-loss-associated physiology, supporting the broader concept that endogenous sleep circuits can be recruited to restore sleep rather than merely suppress behavioral arousal (Kroeger et al., 2018). Likewise, circuit-specific manipulations of preoptic output pathways demonstrate that individual VLPO-associated projections can differentially influence NREM expression, REM-related regulation, autonomic output, and stress-linked physiology (Kostin et al., 2022; Kroeger et al., 2018; Prokofeva et al., 2023; Lu et al., 2002; Zhao et al., 2022). From a translational perspective, these findings are important because they shift therapeutic thinking from global sedation toward network-informed modulation of sleep-promoting circuitry.

At present, however, direct VLPO-targeted therapy in humans remains premature. Current clinical approaches, including non-invasive neurostimulation for insomnia, are not anatomically selective for the VLPO, and any benefit is more reasonably interpreted as modulation of broader sleep-regulatory networks than of a single hypothalamic nucleus (Liu and Li, 2025). Similarly, pharmacological interventions may recruit VLPO-related inhibitory mechanisms indirectly, but the extent to which drug-induced sleep-like states recapitulate physiological VLPO-dependent sleep remains unresolved. Thus, the clinical value of VLPO research currently lies less in defining an immediately targetable anatomical locus than in providing a mechanistic framework for understanding sleep fragmentation, age-related sleep deterioration, and circuit-level therapeutic design.

Future translational progress will depend on linking human neuropathology and neuroimaging to the cell-type and projection-specific organization defined in rodents. Establishing whether human VLPO-homologous neurons share the same molecular markers, relay pathways, and systems-level functions as rodent populations remains essential for rational intervention development (Saper, 2021; Lim et al., 2014). In this sense, the VLPO should be viewed not as a ready-made clinical target, but as a biologically informative entry point for developing more precise treatments for insomnia, neurodegenerative sleep disruption, and disorders of state instability.

### 6.3 Cross-species translation and methodological challenges

Rodent studies have provided foundational insights into VLPO circuitry through lesion experiments, c-Fos mapping, electrophysiology, neural tracing, and opto/chemogenetic manipulation (Sherin et al., 1996; Kostin et al., 2023; Gvilia et al., 2006; Alam et al., 2014; Lu et al., 2000). More recently, transcriptomic and spatial approaches have added a molecular dimension to this framework by revealing multiple inhibitory subclusters and glial-interaction signatures within the preoptic sleep-promoting network (Guo et al., 2020; Guo et al., 2024; Moffitt et al., 2018). However, translating these findings to humans remains challenging. The intermediate hypothalamic nucleus is currently the strongest candidate homolog of the rodent VLPO, yet its anatomical boundaries are less discrete than those in rodents and its functional equivalence—particularly with respect to circadian relay circuitry—remains incompletely defined (Saper, 2021; Lim et al., 2014).

Methodological differences also complicate interpretation. Lesion studies define regional necessity, c-Fos maps activity-associated ensembles, electrophysiology captures state-dependent firing, and transcriptomic analyses identify molecular classes; however, these approaches do not necessarily isolate the same neuronal populations or the same level of organization (Sherin et al., 1996; Gvilia et al., 2006; Alam et al., 2014; Moffitt et al., 2018; Kim et al., 2025; Mickelsen et al., 2019; Romanov et al., 2017; Kim et al., 2019). As a result, discrepancies across studies should not always be interpreted as contradiction, but may instead reflect differences in temporal resolution, sampling strategy, or the distinction between region-level and cell-type-specific analysis. This is especially relevant in the preoptic area, where anatomically adjacent nuclei appear to form distributed and partially redundant sleep-regulatory networks rather than a single anatomically bounded control center.

Cross-species translation is particularly constrained in the case of circadian input pathways. In rodents, direct projections from the SCN to the VLPO appear sparse, with intermediary nuclei such as the DMH and MPOA likely serving major relay roles (Novak and Nunez, 2000; Deurveilher et al., 2002). Whether comparable multisynaptic architectures operate in humans remains unresolved and cannot be assumed on the basis of anatomical proximity alone (Saper, 2021). Bridging this translational gap will require coordinated use of postmortem chemoarchitectural mapping, high-resolution neuroimaging, pharmacological perturbation, and comparative molecular profiling. Until such evidence accumulates, detailed circuit claims are best regarded as strongest in rodents and more inferential in humans.

### 6.4 Physiological and environmental modulators of VLPO-related sleep networks

#### 6.4.1 Sex and reproductive endocrine state

Sex differences represent an important source of physiological variation in sleep–wake regulation and likely influence VLPO-related sleep-promoting circuitry through broader preoptic and hypothalamic mechanisms (Krishnan and Collop, 2006). Epidemiological and experimental evidence indicates sex-dependent variation in sleep architecture, circadian timing, and vulnerability to sleep disruption (Krishnan and Collop, 2006). Estrogen receptor signaling within preoptic regions has been associated with alterations in REM/NREM distribution (Mong and Cusmano, 2016), whereas progesterone metabolites can enhance inhibitory neurotransmission through GABAA receptor modulation, thereby contributing to sedative and sleep-promoting effects (Paul and Purdy, 1992).

During pregnancy and lactation, profound hormonal fluctuations are accompanied by sleep fragmentation and circadian instability, suggesting that reproductive endocrine state can reshape sleep-promoting and arousal-regulating systems, potentially including VLPO-related inhibitory pathways (Mindell et al., 2015). At present, however, direct cell-type-specific evidence for sex-dependent modulation of identified VLPO subpopulations remains limited. The most defensible conclusion is therefore that reproductive endocrine state modulates broader preoptic sleep networks that likely include, but are not restricted to, the VLPO.

#### 6.4.2 Nutritional and metabolic state

Nutritional state is another major modulator of sleep-regulatory circuitry. Negative energy balance, including fasting and starvation, promotes wakefulness in part through activation of hypothalamic arousal systems such as orexin neurons (Yamanaka et al., 2003). Conversely, postprandial and energy-replete states are associated with increased sleep propensity, consistent with earlier sections of this review showing that glucose, leptin-related signals, and orexin-opposed pathways can influence VLPO-related sleep circuitry (Scharbarg et al., 2016; Varin et al., 2015; Ramírez-Plascencia et al., 2022; Yoshida et al., 2006). Chronic metabolic dysregulation—including obesity and overnutrition—is linked to sleep fragmentation and impaired sleep homeostasis in both animal models and humans (Vgontzas et al., 2008).

These findings support a model in which peripheral energy status is integrated across hypothalamic networks and may bias VLPO-related inhibitory output indirectly through glucose-sensitive, leptin-responsive, and orexin-linked pathways rather than through a single dedicated metabolic switch (Saper et al., 2001; Scharbarg et al., 2016; Varin et al., 2015; Ramírez-Plascencia et al., 2022; Yoshida et al., 2006; Yamanaka et al., 2003; Vgontzas et al., 2008). In this sense, VLPO function should be understood as dynamically embedded within organismal energy balance rather than operating independently of broader metabolic state.

#### 6.4.3 Modern lifestyle and circadian disruption

Modern lifestyle factors have introduced sustained environmental pressures on sleep–wake regulation, with likely consequences for VLPO-related sleep circuitry (Saper et al., 2010; Wittmann et al., 2006; Hattar et al., 2002; Contreras et al., 2021). Widespread exposure to artificial light, especially during evening and nighttime hours, alters the photoperiodic signals that normally entrain circadian rhythms. Short-wavelength light from indoor lighting and electronic screens suppresses melatonin secretion, delays circadian phase, and prolongs sleep latency (Saper et al., 2010; Contreras et al., 2021). Light information reaches the SCN via intrinsically photosensitive retinal ganglion cells and the retinohypothalamic tract, thereby influencing downstream preoptic and hypothalamic sleep-regulatory networks (Hattar et al., 2002; Contreras et al., 2021).

Circadian misalignment induced by late-night light exposure, irregular sleep timing, social jetlag, or shift work is therefore likely to disrupt the temporal coordination between SCN output and VLPO-related inhibitory signaling, contributing to fragmented sleep and reduced sleep efficiency (Wittmann et al., 2006; Hattar et al., 2002; Contreras et al., 2021). However, direct circuit-level evidence linking these lifestyle factors to specific VLPO neuronal populations remains limited. The current evidence more strongly supports indirect modulation of broader sleep-regulatory networks through altered melatonin signaling, changed homeostatic sleep pressure, and hypothalamic neuroendocrine imbalance.

### 6.5 VLPO-related vulnerability in neurodegenerative disease

Sleep disturbance is increasingly recognized as both a biomarker and a contributor to neurodegenerative disease, making VLPO-related circuitry highly relevant to disease-associated state instability (Saper, 2021; Lim et al., 2014; Luo et al., 2023; Brown et al., 2010). Age-related degeneration of sleep-promoting hypothalamic neurons is associated with fragmented sleep and reduced slow-wave activity, and postmortem studies have linked neuronal loss in the ventrolateral preoptic/intermediate nucleus region to sleep fragmentation in older adults and in Alzheimer’s disease (Saper, 2021; Lim et al., 2014; Holth et al., 2017). These findings are consistent with the view that degeneration of preoptic inhibitory circuitry contributes to declining sleep continuity during aging and neurodegeneration.

At the same time, the evidence should be interpreted cautiously. Most human data remain region-level and correlative, and do not yet establish causality at the level of defined VLPO subtypes (Saper, 2021; Lim et al., 2014). Moreover, neurodegenerative sleep disruption likely reflects broader network pathology involving circadian, arousal, autonomic, and cortical systems in addition to VLPO-related preoptic populations. Sleep disruption may also exacerbate neurodegeneration by impairing glymphatic clearance of neurotoxic metabolites such as *β*-amyloid and tau (Xie et al., 2013), suggesting a bidirectional relationship in which VLPO-related vulnerability may both reflect and amplify disease progression. Thus, the strongest current interpretation is that VLPO-associated degeneration is one important component of a broader distributed pathology linking sleep instability and cognitive decline.

### 6.6 Current limitations

Despite substantial progress, several limitations continue to constrain interpretation of VLPO function. First, the molecular and functional boundaries of cVLPO and eVLPO remain incompletely resolved, limiting precise assignment of physiological roles to anatomically adjacent preoptic subpopulations (Arrigoni and Fuller, 2022; Saper, 2021; Guo et al., 2020; Guo et al., 2024; Moffitt et al., 2018). Second, the independent and synergistic contributions of GABA and galanin to sleep promotion remain unclear, particularly with respect to target-specific inhibition of arousal nuclei and the relative importance of fast synaptic versus longer-range peptidergic signaling (Kroeger et al., 2018; Kask et al., 1995; Pieribone et al., 1995; Xu et al., 1998; Luppi et al., 1995). Third, the functions of non-galanin GABAergic neurons and glutamatergic VLPO-associated populations remain incompletely defined, especially regarding their downstream targets, state dependence, and roles in rapid transitions, stress-related regulation, or autonomic coupling (Arrigoni and Fuller, 2022; Venner et al., 2019; Vanini et al., 2020; Blumberg et al., 2014).

A further limitation lies in methodological non-equivalence across the literature. Regional lesions, activity markers, electrophysiology, transcriptomics, and projection-specific manipulations each interrogate different levels of organization and do not always converge on identical functional interpretations (Sherin et al., 1996; Moffitt et al., 2018; Mickelsen et al., 2019; Romanov et al., 2017; Kim et al., 2019). Finally, cross-species translation remains limited by uncertainty regarding the precise human homolog of the rodent VLPO and the extent to which rodent-defined circuitry generalizes to human sleep regulation (Saper, 2021; Lim et al., 2014). These limitations argue against overly unitary or VLPO-centric models and support a more cautious interpretation in which the VLPO is viewed as one heterogeneous component of distributed preoptic–hypothalamic–brainstem sleep networks.

### 6.7 Future directions

Future work should prioritize cell-type- and projection-resolved analysis over further expansion of region-level description. Integrating single-cell RNA sequencing with spatial transcriptomics will be essential for defining subtype-specific molecular markers of cVLPO and eVLPO, validating candidates such as P2ry14- and Htr2a-enriched populations, and clarifying how glial, metabolic, and inflammatory signatures map onto functional sleep-promoting circuitry (Guo et al., 2020; Guo et al., 2024; Moffitt et al., 2018; Kim et al., 2025). Intersectional genetic tools and projection-specific manipulations will be required to dissect the independent and synergistic effects of GABA and galanin, and to determine how non-galanin GABAergic neurons and glutamatergic neurons participate in state transitions, autonomic regulation, and stress-linked physiology (Arrigoni and Fuller, 2022; Venner et al., 2019; Vanini et al., 2020; Prokofeva et al., 2023).

Dynamic functional approaches will be equally important. *In vivo* calcium imaging, closed-loop optogenetics, and state-specific recording during natural sleep, sleep deprivation, metabolic challenge, and thermal perturbation should help resolve how VLPO-related populations are recruited across physiological conditions (Kostin et al., 2023; Scharbarg et al., 2016; Varin et al., 2015; Rothhaas and Chung, 2021; Conceição et al., 2019; Kroeger et al., 2018). Comparative work across rodents, non-human primates, and humans—combining neuropathology, molecular mapping, neuroimaging, and pharmacological probes—will be critical for establishing functional homology and improving translational relevance (Saper, 2021; Lim et al., 2014). Finally, cross-nucleus tracing and perturbation studies involving the MnPO, MPOA, parafacial zone, hypothalamic arousal systems, and brainstem REM circuitry should clarify how VLPO-related neurons interact with other sleep-promoting populations within distributed and partially redundant sleep-regulatory networks (Anaclet et al., 2014; Alam et al., 2018; Machado et al., 2022; Anaclet and Fuller, 2017; Novak and Nunez, 2000; Deurveilher et al., 2002; Lu et al., 2002). Progress along these lines will move the field beyond the concept of a unitary sleep center and toward a more precise systems-level model of state regulation.
